# Word and Pseudoword Dictation Impairment in Patients with Primary Progressive Aphasia

**DOI:** 10.1002/alz70857_106419

**Published:** 2025-12-26

**Authors:** Cecilia Coelho Nogueira, Tereza Carvalho Braga, Aline CARVALHO Campanha, Caroline Martins de Araujo, Paulo Caramelli, Karoline Carvalho Carmona, Leonardo Cruz de Souza, Thais Helena Machado

**Affiliations:** ^1^ Universidade Federal de Minas Gerais, Belo Horizonte, Minas Gerais, Brazil; ^2^ Federal University of Minas Gerais, Belo Horizonte, Minas Gerais, Brazil; ^3^ Universidade Federal de Minas Gerais, Belo Horizonte‐MG, Minas Gerais, Brazil; ^4^ Federal University of Minas Gerais, Belo Horizonte, Brazil; ^5^ Universidade Federal de Minas Gerais, Belo Horizonte, Minas ∖gerais, Brazil

## Abstract

**Background:**

Word and pseudoword dictation tasks help characterize errors in patients’ writing, which can aid in diagnosis, classification and provide better guidance for therapeutic strategies in Primary Progressive Aphasia (PPA). Few studies have examined written language in patients with dementia, and even fewer have focused on PPA. The objective of this study was to analyze, classify, and quantify the types of writing errors in adults with different types of PPA using word and pseudoword dictation.

**Method:**

This cross‐sectional study was conducted at a Cognitive and Behavioral Neurology Outpatient Clinic and was approved by the Research Ethics Committee. To date, the study has analyzed 18 patients diagnosed with PPA, using a word and pseudoword dictation protocol. The inclusion criteria required a medical diagnosis of PPA and a minimum of 4 years of schooling. For the word and pseudoword dictation task, errors were classified into various categories, such as neologisms, regularization, graphemic errors, and accentuation errors. Descriptive statistical analysis was performed to report the frequency of the errors.

**Result:**

These preliminary data have 18 patients evaluated, 4 had the semantic type, 4 had non‐fluent/agrammatic variant, 3 had the logopenic variant, and 7 were unclassifiable. All patients were right‐handed, with a mean age of 65 years, an average of 13 years of schooling, and a mean symptom onset of 3.5 years. The types of errors were analyzed by word class. For regular words, errors due to not knowing the rules were the most common (22.8%). For irregular words and pseudowords, graphemic substitution errors were the most prevalent, accounting for 19.1% and 32.4% respectively. It is worth noting that, regardless of word type, 18% of the words were not written due to difficulties in performing the task. Other types of errors occurred with less frequency. We plan to increase the sample size and perform further analysis based on the subtypes of PPA.

**Conclusion:**

In this sample of patients with PPA, errors due to not knowing the rule and graphemic substitution were the most common. Expanding the sample size will allow for a more robust analysis based on the specific aphasia subtypes.

